# Early Steps of Individual Multireceptor Viral Interactions Dissected by High-Density, Multicolor Quantum Dot Mapping in Living Cells

**DOI:** 10.1021/acsnano.4c09085

**Published:** 2024-10-10

**Authors:** Nicolas Mateos, Enric Gutierrez-Martinez, Jessica Angulo-Capel, Irene Carlon-Andres, Sergi Padilla-Parra, Maria F. Garcia-Parajo, Juan A. Torreno-Pina

**Affiliations:** † ICFO—Institut de Ciencies Fotoniques, The Barcelona Institute of Science and Technology, Castelldefels, Barcelona 08860, Spain; ‡ Department of Infectious Diseases, King’s College London, Faculty of Life Sciences & Medicine, London WC2R 2LS, United Kingdom; § Randall Division of Cell and Molecular Biophysics, King’s College London, London WC2R 2LS, United Kingdom; ∥ Division of Structural Biology, Wellcome Centre for Human Genetics, University of Oxford, Oxford OX3 7BN, United Kingdom; ⊥ ICREA, Pg. Lluís Companys 23, Barcelona 08010, Spain

**Keywords:** single molecule localization, quantum dot tracking, single virus tracking, host−pathogen interactions, multimolecular receptor interactions, HIV-1, SARS-CoV-2

## Abstract

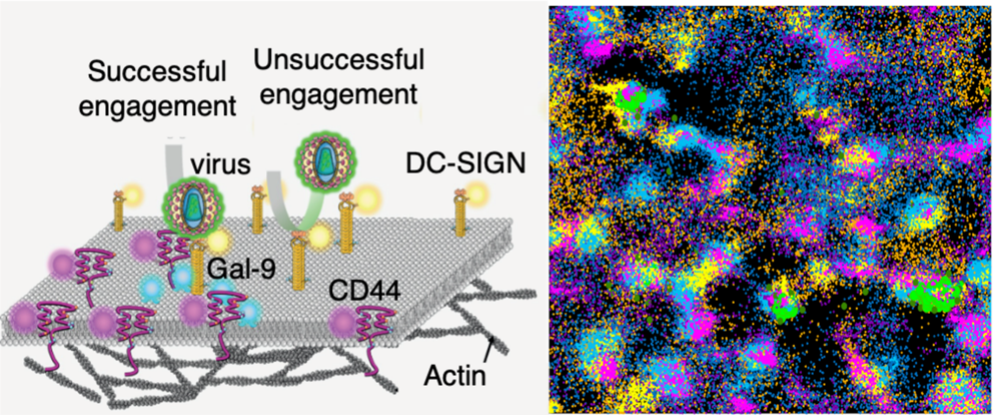

Viral capture and entry to target cells are the first crucial steps that ultimately lead to viral infection. Understanding these events is essential toward the design and development of suitable antiviral drugs and/or vaccines. Viral capture involves dynamic interactions of the virus with specific receptors in the plasma membrane of the target cells. In the last years, single virus tracking has emerged as a powerful approach to assess real time dynamics of viral processes in living cells and their engagement with specific cellular components. However, direct visualization of the early steps of multireceptor viral interactions at the single level has been largely impeded by the technical challenges associated with imaging individual multimolecular systems at relevant spatial (nanometer) and temporal (millisecond) scales. Here, we present a four-color, high-density quantum dot spatiotemporal mapping methodology to capture real-time interactions between individual virus-like-particles (VLPs) and three different viral (co-) receptors on the membrane of primary living immune cells derived from healthy donors. Together with quantitative tools, our approach revealed the existence of a coordinated spatiotemporal diffusion of the three different (co)receptors prior to viral engagement. By varying the temporal-windows of cumulated single-molecule localizations, we discovered that such a concerted diffusion impacts on the residence time of HIV-1 and SARS-CoV-2 VLPs on the host membrane and potential viral infectivity. Overall, our methodology offers the possibility for systematic analysis of the initial steps of viral-host interactions and could be easily implemented for the investigation of other multimolecular systems at the single-molecule level.

Virus-mediated infectious diseases evolving into pandemics pose a recurrent challenge to public health worldwide. Prominent examples are the HIV-1 and SARS-CoV-2 viruses that have resulted in AIDS and COVID-19 pandemics, respectively. As such, huge efforts are being deployed in the development of vaccines and other therapeutic strategies against these, and other viral infections.^1−4^ Understanding the early molecular events leading to viral capture and cell entry is probably the most crucial step toward designing and developing vaccines able to elicit neutralizing antibodies against the different viral strains. Hence, the development of suitable techniques able to capture the early events of viral infections is of urgent need.

Dendritic cells (DCs) are antigen presenting cells of the immune system whose mission is to capture antigens in peripheral tissues and present them to T cells in lymphoid tissues.^5−7^ DCs recognize and capture antigens by expressing a large number of pathogen recognition receptors on their cell membrane. A prominent example is DC-SIGN, a C-type lectin transmembrane receptor expressed on immature DCs (imDCs) able to bind and capture a large plethora of pathogens, including HIV-1.^8^ Although the major targets of HIV-1 infection are CD4^+^-T cells where infection occurs via membrane-fusion,^9^ it has been extensively documented that DCs crucially contribute to HIV-1 infection by influencing viral transmission and CD4^+^-T cell target infection.^8,10,11^ This process is initiated by the capture of HIV-1 by DC-SIGN on imDCs and subsequent DC-SIGN-mediated endocytosis by clathrin-dependent and -independent pathways.^11,12^ Previous work from our group and others showed that basal nanoclustering of DC-SIGN on the plasma membrane of imDCs is crucial for binding and internalization of virus particles, acting as docking sites for HIV-1 to invade the host.^13−15^ Moreover, it has been recently proposed that DC-SIGN also mediates the capture of SARS-CoV-2 viruses^16,17^ although the role for DC-SIGN nanoclustering in SARS-CoV-2 capture and entry is completely unknown. We further showed that galectins, in particular galectin-9 (Gal-9), enhance clathrin-dependent endocytosis of DC-SIGN bound to HIV-1-like particles.^15^ However, whether potential interactions between Gal-9 and DC-SIGN occur prior to and/or during virus capture is not yet known. It is also unknown if additional molecular players at the host cell membrane synergize with DC-SIGN and/or Gal-9 to enhance virus capture and uptake by imDCs.

A prominent technique that has been widely used in the last two decades to study viral infections at the relevant spatiotemporal scales is single virus tracking (SVT).^18−21^ By following the fate of individual viruses, SVT has showed how viruses interact and infect host cells relying on a variety of cellular processes such as membrane-fusion,^22^ clathrin or caveolin-mediated endocytosis^23−28^ and cytoskeleton dependent intracellular transport.^23,29−32^ SVT is typically based on the fluorescence labeling of individual viruses or virus-like-particles (VLP)s and the monitoring of their spatiotemporal behavior in combination with overall fluorescence labeling of the cellular structures of interest. While this approach provides exquisite data of individual viruses or VLPs, the information obtained at the receptors level or from the cellular machinery involved in the uptake and infection process is averaged out by the full labeling strategy used. Approaches that combine SVT together with single particle tracking (SPT) of individual receptors in real time could provide detailed insights into the early events involved in virus-receptor interactions that lead to virus capture and uptake. Unfortunately, detecting a sufficient number of such interaction events for statistical analysis is a daunting task that entails the collection of a large amount of data, as SVT and SPT approaches require stringent sublabeling conditions that when combined together dramatically reduce the probability of capturing individual virus-receptor interactions in real time.

Quantum dots (QD) have been established as a powerful labeling approach to monitor the dynamics of many different types of individual receptors on the plasma membrane of living cells via SPT,^15,33−35^ and also extensively exploited to label different viral components for SVT studies.^15,30,36,37^ This is mainly due to the excellent optical properties of QDs, such as high quantum yield and photostability. In addition, their broad excitation spectra and narrow emission spectra make QDs ideally suited for multicolor imaging and SPT applications.^31,38,39^ Indeed, real time interactions between two similar receptors have been monitored by dual color SPT,^15,33,35^ but extending this approach to study interactions involving more than two labeled receptors is challenged by the low labeling densities needed for conventional SPT.^40^

Here we used QDs to label three different membrane components of imDCs derived from healthy donors and combined it with GFP-labeling of HIV-1- or SARS-CoV-VLPs to resolve simultaneous multimolecular interactions at the single level in real time. In contrast to standard SPT, our strategy relies on high-density labeling of QDs combined with multicolor single molecule localization determination in real time in order to construct spatiotemporal localization maps without the need of reconnecting individual localizations. Such maps significantly boost the observation of multimolecular interactions, thus increasing statistical throughput while retaining the advantages of standard SPT in terms of spatiotemporal resolution and single molecule detection sensitivity. Using this approach, we revealed dynamic interactions between three different membrane components on imDCs prior to VLP engagement. Furthermore, we found that nanometer-scale colocalization of all three proteins enhanced DC-SIGN-mediated successful capture of HIV-1- and SARS-CoV-2-VLPs.

## Results

### Generation of Multicolor High-Density Maps from Single QDs Imaging Data

Our method consists on live cell imaging and spatial localization over time of individual QDs having different emission spectra and attached to the molecules of interest. Crucially different from standard SPT, we increase the labeling density (typically 2 orders of magnitude higher than for SPT), while working at subsaturating conditions so that individual QDs are resolved in each diffraction-limited image. We then retrieve the localization positions of the QDs with nanometric precision on a frame-to-frame basis and reconstruct multicolor high-density localization maps (HiDenMaps) of the space explored by the different molecules as a function of time.

**Figure 1:**
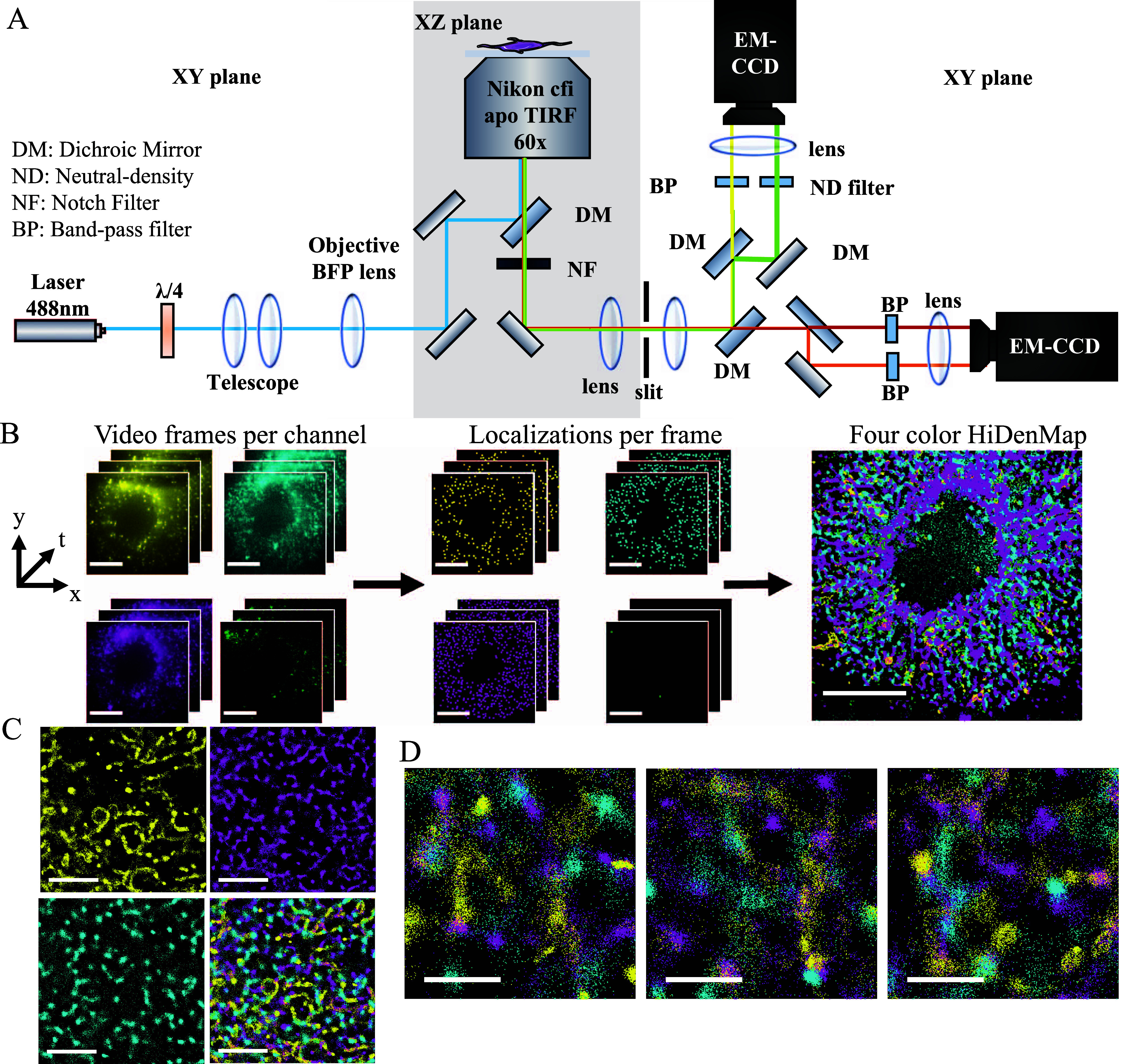
Generation of HiDenMaps from high-density multicolor single molecule imaging in living cells. (A) Schematics of the custom-built four-color TIRF inverted microscope. The sample is illuminated by a 488 nm laser line focused on the back focal plane of the objective. The fluorescence emission is collected through the objective and split into four optical paths using dichroic mirrors and filtered using appropriate band-pass filters. Each emission is focused on two different regions of an EM-CCD camera, leading to a four-color detection scheme. (B) Schematics showing the generation of four-color HiDenMaps. From left to right: Video frames for each of the four channels. For each channel individual QDs and VLPs are localized at each frame of the video (middle panel). The localizations per frame and channel are then transformed using an affine transformation to correct for the aberration of the optical paths and collapsed into a single image (right panel). The localization precision of each individual QD resulted in 18.3, 9.86, and 19.4 nm for QD605, QD655, and QD705, respectively, and 27.7 nm for the VLPs. Scale bars, 10 μm. (C, D) Representative three-color HiDenMaps with an integration time of 30 s. (C) DC-SIGN (yellow), CD44 (magenta), Gal-9 (cyan) and overlay of the tripartite proteins (lower right panel). Scale bars, 10 μm. (D) Representative enlarged overlay regions of the tripartite proteins on different regions of the cell membrane. Scale bars, 1 μm.

To simultaneously image individual VLPs and QDs bound to different molecules on the membrane of imDCs we designed a multicolor single molecule-sensitive inverted microscope working in a total internal reflection fluorescence (TIRF) configuration (Figure 1A and Supporting Information, four-color single molecule sensitive microscope). Since QDs have a broad excitation spectra, we used a single laser line (λ_exc_ = 488 nm) for the excitation of three different QDs (QD605, QD655, and QD705) and tagged VLPs with a green fluorescent protein (eGFP-Gag), allowing simultaneous four-color excitation with a single laser, avoiding chromatic aberrations and simplifying the excitation path. The four color fluorescence emission was collected through a Nikon CFI APO TIRF 60× objective, split into four optical paths and directed to two different EM-CCD cameras, each of them recording two different spectral ranges (Figure 1A). The crosstalk between the different fluorescence channels ranged between 5 and 10% allowing multicolor imaging with minimal color crosstalk (Figure S1).

To study early events of viral capture on imDCs, we focused on DC-SIGN as the primary membrane receptor for HIV-1 binding on imDCs. Moreover, we previously showed that Gal-9, a tandem repeat galectin, influenced clathrin-dependent endocytosis of DC-SIGN bound to HIV-1-VLPs.^15^ In addition, copatching experiments showed large colocalization between Gal-9 and CD44, a heavily glycosylated transmembrane protein, and between DC-SIGN and CD44, suggesting that interactions between DC-SIGN and Gal-9 might be coordinated by CD44.^15^ Interactions between Gal-9 and CD44 have been previously reported on T cells and NK cells,^41−43^ and proteomic analysis showed that DC-SIGN, CD44 and Gal-9 are contained on imDCs phagosomes.^44^ Therefore, we set to investigate potential interactions between the tripartite proteins: DC-SIGN, CD44, and Gal-9, at the single molecular level and their impact on the initial steps of virus binding. For this, we immunolabeled DC-SIGN and CD44 using single chain antibodies (Abs) to prevent Ab cross-linking (see Experimental Methods section). Biotinylated single chains Abs were then conjugated to streptavidin-coated QDs (QD655 and QD705, for DC-SIGN and CD44, respectively) in an excess of free biotin to block unoccupied streptavidin sites, preventing cross-linking and ensuring that only one QD is attached to the protein of interest, following a similar strategy as earlier reported^15,35,45^ (see also Experimental Methods section and Figure S2). In addition, we tagged recombinant human Gal-9 using a biotin–streptavidin-QD (QD605) labeling scheme (see Experimental Methods). Multicolor QD imaging was performed at 30 nM labeling conditions for the three different proteins, which is around 2 orders of magnitude higher than typically used for standard SPT experiments.^15^

To generate multicolor HiDenMaps from the recorded videos, we localized individual QDs with the FIJI plugin Trackmate at each video frame of each channel and collapsed all the localizations into a single image using MATLAB^46^ (Figure 1B). To account for variations in the expression level of each of the proteins due to cell-to-cell variability but also donor-to-donor variability, we normalized the number of localizations for every single HiDenMap (Supporting Information, normalization of the localizations of HiDenMaps). As a three-color example, we recorded a 90 s multicolor fluorescence video of DC-SIGN, CD44 and Gal-9 on the cell membrane of imDCs using a camera framerate of 30 Hz (Videos S1, S2, S3, and S4). HiDenMaps generated by collapsing all the single molecule localizations for each of the channels during 30 s reveal that the three proteins dynamically explore the cell membrane in a rather heterogeneous fashion, describing 2D patterns in their lateral diffusion (Figure 1C and Video S5). Moreover, when visually observing in greater detail (3 μm × 3 μm zoom-in regions), it appears that the three proteins explore similar space in contiguous or even overlapping regions of the cell membrane (Figure 1D and Video S5). These observations hint to the existence of some type of correlated spatial (and/or temporal) organization of the tripartite proteins at the mesoscale (i.e., >0.5 μm).

### HiDenMaps Reveal that DC-SIGN, CD44, and Gal-9 Explore the Environment in an Interconnected Manner

**Figure 2:**
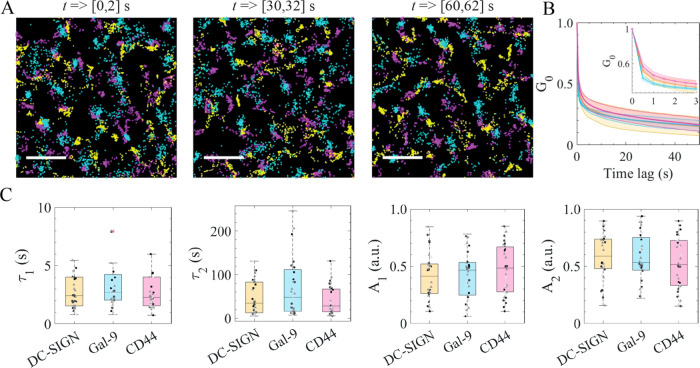
Temporal evolution of multicolor HiDenMaps for the tripartite proteins. (A) Representative 2 s time windows of three-color HiDenMaps at different observation times. Yellow corresponds to DC-SIGN, magenta to CD44 and cyan to Gal-9. The imaging was performed at 30 Hz and the total acquisition was 90 s. Scale bars, 2 μm. (B) Temporal autocorrelation decay curves for DC-SIGN, CD44 and Gal-9 with a Δ*t* of 500 ms. The weighted line corresponds to the mean between donors and the shaded area to the standard deviation between donors. The inset corresponds to the first three seconds of the autocorrelation curves. (C) The parameters (τ_1_, τ_2_, *A*_1_, *A*_2_) fitted from the autocorrelation decays for DC-SIGN, Gal-9, and CD44. The whiskers represent the quartiles and the median value for each distribution. Each point in the scatter plot corresponds to a single cell. The data shown in (B, C) corresponds to three donors. For each donor we measured two samples, 10 cells per sample and roughly 8 ROIs per cell. Moreover, each donor was measured on a different day. The Kruskal–Wallis statistical test was performed and no statistical differences were found between the different proteins.

Since HiDenMaps encode both spatial and temporal information, we first extracted the relevant temporal scales by generating three-color HiDenMaps of DC-SIGN, CD44 and Gal-9 at different time windows, and accumulating the localizations during 2 s per window (see Experimental Methods section). Similar to the previously generated maps built up with all the localizations (Figure 1C,D), we observed contiguous spatial exploration of the tripartite proteins in all temporal windows (Figure 2A), suggesting a coordinated diffusion over time. To quantify the degree of temporal correlation we then used an overlapping sliding time-window of Δ*t* = 500 ms to temporally accumulate localizations and computed the autocorrelation curves as a function of time for the three proteins (Figure 2B). The length of the window results from a trade-off of having to accumulate a sufficient number of localizations while retaining high-temporal resolution. The autocorrelation curves were best fitted using a double exponential function from which we extracted two characteristic decay time components (τ_1_,τ_2_) as well as the respective amplitudes, *A*_1_ and *A*_2_ (see Experimental Methods section and extended explanations in the Supporting Information, spatiotemporal autocorrelation decay curves). When we applied this type of analysis to the generated HiDenMaps of DC-SIGN, CD44, and Gal-9, we obtained comparable τ and *A* values for the three proteins (median values for τ_1_ ≈ 3 s; τ_2_ ≈ 30 s; *A*_1_*≈* 0.4 and *A*_2_ ≈ 0.6) (Figure 2C). The similarities in decay times and amplitudes of the autocorrelation curves confirm that the three proteins temporally explore their surrounding in a highly coordinated manner.

We next asked whether this “inter-connection” at the mesoscale is already established at the nanoscale. To address this question, we performed SPT of DC-SIGN, Gal-9, and CD44 using a reduced labeling density (1 nM) as compared to that used for the generation of HiDenMaps. This approach allowed the reconnection of individual trajectories over nanoscale regions of the membrane at shorter temporal scales. Individual trajectories of the three proteins were analyzed by generating plots of the mean squared displacement as a function of time lag, from which the short-term diffusion coefficients *D*_1–4_ were calculated from the initial slopes by linear fit from the first to the fourth point (Figure S3). The extracted *D*_1–4_ histograms for DC-SIGN and CD44 were comparable to previous reported values using organic dyes^45,47^ further confirming that our QD labeling strategy does not influence the diffusion of the proteins (we note that to the best of our knowledge, no direct measurements of Gal-9 mobility have been reported in the literature). We observed large differences in the distribution of the *D*_1–4_ values for DC-SIGN, CD44 and Gal-9 (Figure S3), indicating unrelated diffusion at the nanoscale. Together, these results thus show that whereas at the nanoscale each protein diffuses and explores its nanoenvironment with different mobilities, a high degree of coordinated exploration exists over longer temporal and spatial scales.

### Simultaneous Three-Color SPT and SVT Reveals Single HIV-1 VLP Capture Events by the Tripartite Proteins

**Figure 3:**
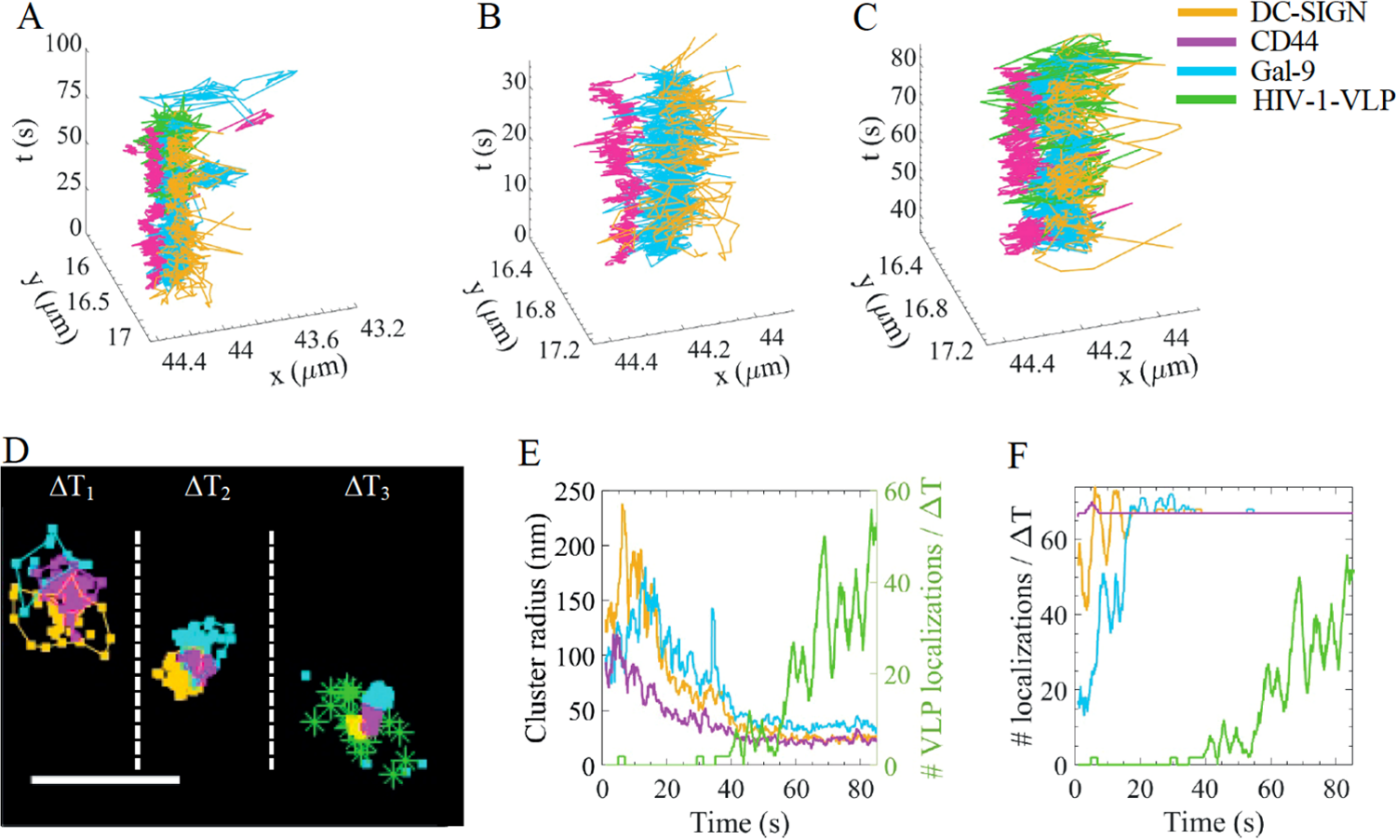
Temporal tracking of single HIV-1 VLP engagement by the tripartite proteins. (A) Single trajectories of DC-SIGN (yellow), CD44 (magenta), Gal-9 (cyan), and HIV-1 VLP (green) as a function of time. (B, C) Enlarged temporal windows of (A), prior to (B) and during VLP engagement (C). (D) Snapshots of three representative temporal windows showing accumulated localizations (during 2 s) of the positions explored by DC-SIGN, CD44, and Gal-9, prior to (Δ*T*_1_, Δ*T*_2_) and during VLP engagement (Δ*T*_3_). The connecting lines between the symbols correspond to the identification and tracking of clusters of localizations for each of the proteins. The three proteins diffuse contiguous to each other prior to VLP engagement and their spatial proximity increases during VLP engagement. The three time-windows shown are Δ*T*_1_ ∈ [2, 4] s, Δ*T*_2_ ∈ [25, 27]s and Δ*T*_3_ ∈ [70, 72] s. Scale bar, 500 nm. (E) Quantification of cluster radius (left axis) and the number of VLP localizations per time-window (2 s) (right axis) as a function of time. (F) Number of localizations per cluster and time-window (2 s) for the three proteins and for the HIV-1 VLP. In (E, F), the data for the number of HIV-1 VLP localizations is the same.

To address if the spatiotemporal coordination of the tripartite proteins plays a role on the capture of HIV-1 VLPs on imDCs, we first performed multicolor SPT of the three proteins in combination with SVT of a eGFP-Gag-labeled HIV-1 VLP decorated with the BL4–3 envelope glycoprotein (Env), the ligand of DC-SIGN (see Experimental Methods section for details on the VLPs generation). We focused on events where we detected the presence of HIV-1 VLPs together with the simultaneous occurrence of trajectories of the three proteins in the neighborhood of the HIV-1 VLPs, and followed their evolution in time (Figure 3A). Given the low labeling density of standard multicolor SPT, the probability of capturing such events is extremely small requiring hundreds of hours of analysis for their detection. Consistent with the HiDenMaps, tracking of the individual trajectories showed contiguous diffusion of the three proteins over time *prior* to HIV-1 VLP engagement (Figure 3A,B and Video S6). Notably, initial HIV-1 VLP capture in the close vicinity of the tripartite proteins occurred in an intermittent fashion, with multiple binding and unbinding events that finally resulted in a more stable binding and coincided with an increase in the spatial proximity of the three proteins (Figure 3C).

To statistically quantify these observations, we turned to HiDenMaps which significantly increase the probability of detecting multimolecular events as compared to SPT. We generated temporal windows of 2 s and overlaid the single molecule localizations of the three proteins and of the HIV-1 VLP into single maps (Figure 3D and Video S7). To estimate the effective region explored by each of the proteins as a function of time, prior and during HIV-1 VLP engagement, we applied a DBSCAN cluster algorithm,^48^ commonly used for cluster analysis of single molecule localization super-resolution images^49−52^ (see Experimental Methods section). This analysis showed a clear and gradual reduction in the cluster radius of the three different proteins during the capture event indicative of their increased spatial proximity together with a concomitant and progressive increase in the number of detected HIV-1 VLP localizations (Figure 3E and Video S7). Multicolor HiDenMaps reveal that HIV-1 VLP binding occurs on regions of the cell membrane already pre-enriched with the tripartite proteins and that their proximity is increased during viral engagement, enhancing in turn viral binding strength. To further strengthen these results we analyzed the number of localizations per protein cluster during viral capture. Indeed, whereas for DC-SIGN, CD44, and Gal-9 the number of localizations remained constant over time, indicating stable engagement of the tripartite proteins during HIV-1 VLP capture, the number of HIV-1 VLP localizations increased over time, consistent with virus binding stabilization (Figure 3F). As a whole, these results suggest that the spatiotemporal coordination of the tripartite proteins prior to virus engagement is an important step for the successful capture of HIV-1 VLPs, and moreover, that the binding strength of the virus is directly related to the spatiotemporal residence of the tripartite proteins at the virus-docking site.

### Four-Color HiDenMaps Reveal Enhanced Interactions of HIV-1 and SARS-CoV-2 VLPs with the Tripartite Protein Nanoplatforms

**Figure 4:**
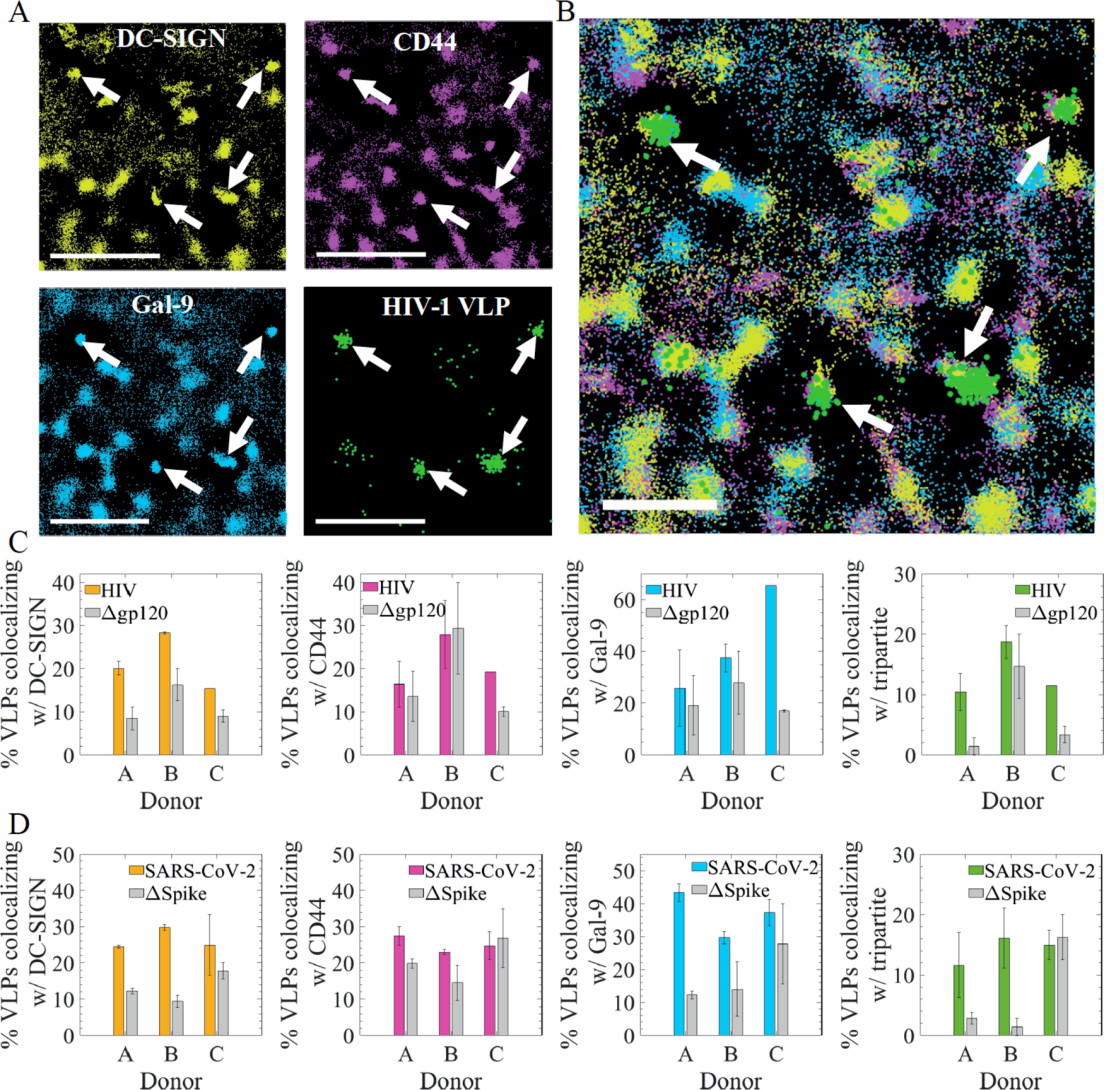
HIV-1 and SARS-CoV-2 VLPs exhibit enhanced spatiotemporal colocalization with the tripartite nanoplatforms. (A) Representative four-color HiDenMaps of DC-SIGN (yellow), CD44 (magenta), Gal-9 (cyan) and HIV-1 VLP (green), generated from accumulating localizations during 50 s. White arrows in the different panels highlight multiple occurrences of simultaneous spatial colocalization of the tripartite proteins and the HIV-1 VLPs. Scale bars, 1 μm. (B) Overlayed HiDenMap of the different panels shown in (A). (C) Percentage of HIV-1 VLPs (colored bars) colocalizing with (from left to right): DC-SIGN, CD44, Gal-9 and with all three proteins, for three different donors investigated. Results obtained with the mock virus (Δgp120) are included as gray bars. (D) Similar as (C) but for SARS-CoV-2 VLPs (colored bars) and mock virus (ΔSpike) (gray bars). Total number of HIV-1 VLPs detected: 64 for donor A, 53 for donor B, and 26 for donor C. Total number of Δgp120 VLPs detected: 88 for donor A, 52 for donor B, and 216 for donor C. Number of cells analyzed for the HIV-1 VLP experiments: 40 for donor A, 40 for donor B, and 30 for donor C. Total number of SARS-CoV-2 VLPs detected: 132 for donor A, 74 for donor B, and 87 for donor C. Total number of ΔSpike VLPs detected: 106 for donor A, 88 for donor B, and 52 for donor C. Number of cells analyzed for the SARS-CoV-2 VLPs experiments: 40 for donor A, 40 for donor B, and 40 for donor C. The bars represent the standard deviation between two independent experiments.

To statistically enquire on the different spatiotemporal interactions between the tripartite proteins in the context of virus engagement we generated HiDenMaps for each of the three proteins and for the HIV-1 VLPs (Figure 4A) and overlaid all the localizations in a single map (Figure 4B). Visual inspection of such maps showed hotspots of enriched localizations for each of the proteins, forming distinct nanoplatforms on the cell membrane that spatially overlap with each other (white arrows in Figure 4A,B). Indeed, we observed a high degree of colocalization events between HIV-1 VLPs and the three protein nanoplatforms (white arrows in Figure 4A,B). In strong contrast to standard SPT approaches where the probability of observing such events is vanishing small, HiDenMaps are able to uncover multiple virus-receptor interactions at the single molecule level, underscoring its effectiveness for monitoring multimolecular interactions in real time.

To quantify these observations we identified clusters of localizations for all the four different components using DBSCAN and performed colocalization analysis between HIV-1 VLPs and the different proteins (Supporting Information, multicolor colocalization algorithm). As control, we performed similar experiments and colocalization analysis using mock HIV-1 VLPs lacking the Env protein. Moreover, to account for variations in the expression level of each of the proteins due to cell-to-cell variability and donor-to-donor variability, we performed experiments on multiple monocyte derived imDCs from three different healthy donors. We obtained a significant increased (15–30%) colocalization between DC-SIGN and HIV-1 VLPs as compared to mock VLPs in all donors analyzed (Figure 4C, left panel) consistent with the fact that DC-SIGN is the main receptor for HIV-1 on imDCs.^8,15^ In the case of CD44 and Gal-9, only one donor (Donor C) exhibited increased colocalization with HIV-1 VLPs as compared to mock VLPs (Figure 4C, middle panels). When analyzing the three proteins together, a high colocalization with HIV-1 VLPs above that of mock viruses was obtained in two out of the three donors analyzed (Figure 4C, right panel). To further confirm that the measured colocalization between the tripartite proteins and HIV-1 VLPs is real and not the result of stochastic binding of the viruses to these regions, we also compared the experimental data to *in silico* experiments of HIV-1 VLPs randomly landing on experimentally obtained HiDenMaps of the three different proteins on imDCs that have not been exposed to the VPLs. A low degree of colocalization between the tripartite proteins and the randomly distributed HIV-1 VLPs was obtained and comparable to that of the mock viruses (Figure S4) indicating that the measured colocalization between the tripartite proteins and HIV-1 VLPs is real. Together, these results reveal enhanced interaction of HIV-1-VLPs with predocking nanoplatforms formed by DC-SIGN, CD44, and Gal-9.

We then asked whether our methodology could be extended and generalized to the study of other virus-receptor interactions. Considering the urgence associated with the global COVID-19 pandemics, we applied our experimental settings to study SARS-CoV-2 VLPs capture by imDCs. Recent reports have identified DC-SIGN as a receptor for the SARS-CoV-2 virus, although the entry mechanisms remain to be elucidated.^16,53^ As DC-SIGN acts in concert with CD44 and Gal-9 to increase the binding of HIV-1 VLPs, we enquired whether a similar molecular mechanism could operate on the DC-SIGN-mediated binding of SARS-CoV-2. In order to generate SARS-CoV-2 VLPs, a plasmid encoding the S-protein ([D614G] the Wuhan variant with a point mutation) was expressed using the HIV-1 VLP backbone (see Methods). The same mock virus without any receptor specific membrane protein, i.e., ΔSpike, was used as a negative control. More than 25% of SARS-CoV-2 VLPs colocalized with DC-SIGN in the three donors analyzed, significantly above that of the Δ*S*pike mutant (Figure 4D, left panel), confirming that DC-SIGN is indeed a receptor for SARS-CoV-2 VLPs. Increased colocalization of SARS-CoV-2 VLPs with CD44 or Gal-9 was only observed in one of the donors (Figure 4D, middle panels). Similar to HIV-1 VLPs, enhanced colocalization of SARS-CoV-2 VLPs with the tripartite proteins was observed in two out of the three donors, as compared to experiments performed using ΔSpike VLPs (Figure 4D, right panel). Thus, these results strongly indicate that predocking nanoplatforms formed by the tripartite proteins favor the interaction of DC-SIGN, not only with HIV-1, but also with SARS-CoV-2.

### DC-SIGN, CD44, and Gal-9 Predocking Nanoplatforms Enhance Viral Capture on the Cell Membrane of imDCs

**Figure 5:**
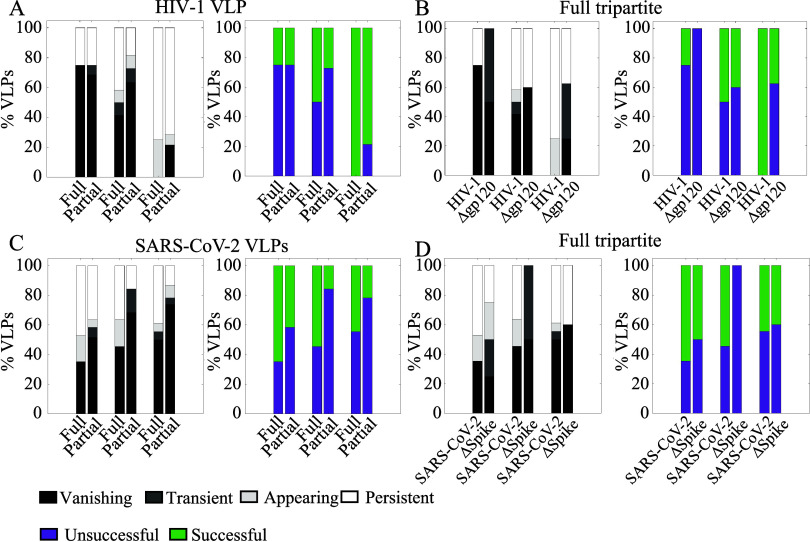
HIV-1 and SARS-CoV-2 VLPs show enhanced viral engagements on the cell membrane upon simultaneous binding to the tripartite nanoplatforms. (A) left panel: Percentage of HIV-1 VLPs showing different engagement dynamics (vanishing, transient, appearing and persistent) when they fully colocalize with all three proteins (DC-SIGN, CD44, and Gal-9) i.e., denoted as “Full” in the plot, or with only a subset of the proteins, denoted as “Partial”. Right panel: Percentage of successful (green) and unsuccessful (violet) HIV-1 VLP engagements corresponding to the left panel. Data are shown for three different donors (from left to right: Donors A, B, C). (B) left panel: Dynamic engagement for VLPs colocalizing with all three proteins, for HIV-1 VLP and the mock virus Δgp120 VLPs. Right panel: Percentage of successful (green) and unsuccessful (violet) VLP engagements corresponding to the left panel. Data are shown for three different donors (bar-sets from left to right: Donors A, B, C). (C) Similar to (A) but for SARS-CoV-2 VLP. Data are shown for three different donors (bar-sets from left to right: Donors A, B, C). (D) Similar to (B) but for SARS-CoV-2 VLP and the mock virus ΔSpike. Data are shown for three different donors (bar-sets from left to right: Donors A, B, C). Total number of HIV-1 VLPs detected: 64 for donor A, 53 for donor B and 26 for donor C. Total number of Δgp120 VLPs detected: 88 for donor A, 52 for donor B and 216 for donor C. Number of cells analyzed for the HIV-1 VLP experiments: 40 for donor A, 40 for donor B and 30 for donor C. Total number of SARS-CoV-2 VLPs detected: 132 for donor A, 74 for donor B and 87 for donor C. Total number of ΔSpike VLPs detected: 106 for donor A, 88 for donor B and 52 for donor C. Number of cells analyzed for the SARS-CoV-2 VLP experiments: 40 for donor A, 40 for donor B and 40 for donor C.

Finally, we assessed whether the enhanced interaction of the tripartite nanoplatforms with HIV-1 and SARS-CoV-2 VLPs results in increased viral capture. To address this question, the lateral behavior of individual viruses was analyzed and correlated with their interaction probability with the tripartite proteins. In other words, HiDenMaps of the different VLPs built up during 90 s observation times were generated and classified according to whether: (I) the VLP was engaged on the cell membrane before the acquisition started but vanished from the cell membrane before the end of the acquisition time. This behavior was classified as “vanishing” (Video S8); (II) the VLP appeared and vanished during the acquisition time, being classified as “transient” (Video S9); (III) the VLP appeared during the acquisition time and remained engaged on the cell membrane, being classified as “appearing” (Video S10) and finally; (IV) the VLP remained engaged during the whole acquisition time. This behavior was classified as “persistent” (Video S11). Representative examples of HiDenMaps from each category are shown in Figure S5. We then correlated these different VLPs behaviors to their full colocalization with the tripartite proteins, or to a partial colocalization, i.e., when the VLP colocalizes with only one or two of the tripartite proteins. In two out of the three donors we computed a higher percentage of appearing and persistent HIV-1 VLP events coinciding with a full colocalization of the three proteins (Figure 5A, left), whereas a lower percentage of these events were obtained when the HIV-1 VLPs partially colocalized with only one or two of the other proteins (i.e., partial) (Figure 5A, left). Since the residence time of the VLPs on the cell membrane likely reflects the strength of viral engagement, we further grouped transient and vanishing VLPs behaviors and classified them as unsuccessful viral capture events whereas appearing and persisting behaviors were classified as successful events. Higher successful engagement of HIV-1 VLPs was observed when the three proteins fully colocalized with the virus in two of the three donors, as compared to VLPs that partially colocalized with only one or two of the proteins (Figure 5A, right). These results imply that binding of HIV-1 VLPs by the three proteins increases the probability of virus capture on the cell membrane of imDCs. Moreover, successful viral capture by the tripartite proteins was higher for the HIV-1 VLPs as compared to VLPs lacking the envelope protein (BL4–3) for all three donors (Figure 5B, HIV-1 vs Δgp120), confirming once more the specificity of DC-SIGN in HIV-1 viral capture.

Finally, we performed the same type of analysis with SARS-CoV-2 VLPs and also found an increased successful engagement of SARS-CoV-2 VLPs with the three proteins together (Figure 5C, full vs partial) in all three donors. Moreover, the involvement of DC-SIGN in the processes of successful SARS-CoV-2 VLPs capture was also confirmed, as higher successful engagement events by the tripartite proteins were observed on the full VLPs as compared to mock viruses lacking the Spike protein (Figure 5D, SARS-CoV-2 vs ΔSpike). Altogether, these results robustly show that preformed docking nanoplatforms of DC-SIGN, CD44 and Gal-9 enhance viral capture on the cell membrane of imDCs.

## Discussion

The multicolor HiDenMap methodology described here provides a tool toward the understanding of multimolecular interactions at the single molecule level in living cells. As an example, we have combined multicolor HiDenMaps of viral receptors on the cell membrane of imDCs derived from healthy donors with HiDenMaps of single viruses thereby visualizing multiple receptor-viral engagements in real time and at the single molecule level. In stark contrast to standard SPT experiments that generate trajectories of the motion of individual molecules by connecting their center of mass position over time, HiDenMaps collects the molecular positions over arbitrary time windows. Since HiDenMaps do not require trajectories reconnection, higher labeling conditions can be used, hence increasing data throughput and enabling the identification of multiple virus-receptor interactions in a single experiment.

Using this methodology, we simultaneously captured the spatiotemporal organization of DC-SIGN, a viral receptor of HIV-1 and SARS-CoV-2 viruses in imDCs, and of CD44 together with Gal-9. We discovered that these three proteins dynamically explore the cell membrane in a highly coordinated and synchronous manner. Such a coordinated diffusion is likely orchestrated by the cortical actin cytoskeleton and by the transient interactions (either directly or indirectly) of any of these proteins to actin. Indeed, the temporal scales retrieved from our analysis (∼3 and ∼30 s) are consistent with the cortical actin acting as a master regulator on the spatiotemporal organization of the cell membrane.^54^ Moreover, CD44 has been proposed to act as a picket protein that links the underlying actin cytoskeleton and the extracellular milieu^45,47^ and previous results from our group showed the dependence of Gal-9 on DC-SIGN diffusion and its lateral organization on the cell membrane.^15^ With the data provided by the simultaneous HiDenMaps of the tripartite proteins we now postulate that Gal-9 might serve as a linkage to connect DC-SIGN to CD44, so that the concerted motion of the tripartite proteins is ultimately guided by the direct interaction of CD44 to actin.

We further addressed the relevance of these tripartite nanoplatforms and their coordinated spatiotemporal diffusion on the capture of HIV-1 and SARS-CoV-2 VLPs by introducing an additional GFP-tagged VLP fluorescence channel, extending our setup to a four color configuration. We resolved individual interactions between VLPs and DC-SIGN and uncovered the relevance of CD44 and Gal-9 forming basal nanoplatforms prior to virus engagement and fully overlapping with DC-SIGN. This coordinated spatiotemporal organization resulted crucial for the successful engagement of both HIV-1 and SARS-CoV-2 VLPs. Indeed, we found an increased successful engagement of HIV-1 VLPs when the viruses colocalized with the tripartite proteins indicating that predocking nanoplatforms of the three proteins promote a more efficient viral capture. We also applied our methodology to SARS-CoV-2 VLPs. Since it has been recently reported that DC-SIGN is a receptor for SARS-CoV-2 viruses,^16,53^ we hypothesized that SARS-CoV-2 capture could also be mediated by the tripartite proteins on imDCs. Indeed, our results showed increased binding of SARS-CoV-2 viruses to the tripartite nanoplatforms as compared to mock viruses. These results thus suggest a potential generalized mechanism of virus capture being mediated by DC-SIGN/CD44/Gal-9 nanoplatforms on the membrane of imDCs. The crucial involvement of CD44 and Gal-9 in this process could contribute to the development of antiviral therapeutic strategies targeting any of these two proteins.

## Conclusions

In summary, the methodology reported here brings an advance to the field by enabling the study of multimolecular interactions at the single molecule level, in real time while retaining all the advantages of single molecule imaging methods. We have applied this methodology to follow the dynamics of individual viruses in combination with up to three different proteins on the cell membrane of primary cells but it can certainly be extended to the study of other multicomponent processes in the cell. As such, our experimental approach enables the quantitative study of multimolecular dynamic processes at relevant spatiotemporal scales with the potential of uncovering phenomena that have remained inaccessible until now given the limitations of current single molecule imaging methods.

## Experimental Methods

### Primary Cell Culture

Human immature dendritic cells (imDCs) were obtained from peripheral blood mononuclear cells (PBMC) from HIV-1 seronegative donors using a Ficoll-Hypaque gradient (Alere Technologies AS). The monocyte population was selected by adherence on a T75 cm^2^ flask for 1 h. imDCs were obtained by culturing the monocytes in complete RPMI with 1.000 IU/mL (granulocyte-macrophage colony-stimulating factor (GM-CSF)) and IL-4 (interleukin-4) both from R&D for 6 days. The medium was replaced every 2 days with fresh GM-CSF and IL-4. Experiments were performed at day 6 from the monocyte extraction.

### Antibodies and Reagents

Monoclonal mouse antihuman CD44 (Clone G44–26) and monoclonal mouse anti-CD209 (Clone DCN46) were obtained from BD Biosciences. Recombinant human Gal-9 protein (Cat. number 9064-GA) was obtained from R&D systems. SARS-CoV-2 Spike Protein (RBD) Chimeric Recombinant Rabbit Monoclonal Antibody (P05DHuRb) tagged with Alexa Fluor 647 was obtained from eBioscience. Streptavidin QDs (565, 605, 655, and 705) were obtained from Thermo Fisher scientific. These QDs are based on a CdSe core nanocrystal surrounded by a ZnS shell to improve the brightness and stability of the QDs. Moreover they are functionalized with streptavidin enabling the attachment of the QDs to the biomolecules of interest (DC-SIGN, CD44 and Gal-9).

### Single Chain Antibody Generation

All the experiments reported here have been performed using single chain antibodies to label DC-SIGN and CD44 in order to prevent antibody cross-linking (see Figure S2A for the general labeling strategy used for the generation of SPT and HiDenMaps). Both antihuman CD209 and CD44 single chain antibodies were generated using a similar protocol. First, the full chain antibodies were dialyzed using 10K dialysis devices (Thermo Scientific Slide-A-Lyzer MINI Dialysis Devices, 10 K MWCO) against PBS for 8 h at 4 °C. Second, we concentrated the dialyzed full chain antibodies to a concentration of 1 mg/mL. We then reduced the antibodies using DTT (1,4-dithiotrheritol, Sigma-Aldrich) at 1 mM, let the mixture to reduce at room temperature for 1 h while rotating and after that dialyzed overnight against PBS using the 10 K dialysis devices at 4 °C. We stabilized the broken sulfide-bonds with Iodoacetamide at 20 mM. We let the mix at room temperature for 1 h rotating gently and dialyzed to remove excess iodoacetamide overnight at 4 °C. Figure S2B shows the electrophoresis gel for the generated single chain antibodies together with the full chain antibodies as a control.

### Biotinylation and Conjugation to Quantum Dots

We performed the biotinylation of single chain antibodies and of Gal-9 with EZ-Link Sulfo-NHS-LC-Biotin (Thermo Fisher). For this, we added a 20× mol excess of biotin and let the mixture to shake for 1 h in ice. Then, we dialyzed using 10 K units overnight at 4 °C to remove excess of nonreacting biotin. To conjugate the biotinylated single chains and Gal-9 to the QDs, we mixed equal ratios of single-chain/Gal-9 and QDs in 5× excess of free biotin. The competition with excess of free biotin ensures a 1:1 labeling ratio between the target protein and the QD, as earlier reported.^15,35^ To obtain a target concentration of 300 nM of stock conjugates, we first mixed 300 nM of QDs with 1.5 μM biotin and then added 300 nM of single-chain/Gal-9. Importantly, to avoid artifacts due to cross-linking of the recombinant added Gal-9 proteins, titrations were performed until single molecules of the conjugate Gal-9/QD were detected.

### Labeling Strategy

For SPT experiments, we used conjugates of Gal-9/QD565, α-CD44/QD655, and α-DC-SIGN/QD705 at a concentration of 1 nM. For the HiDenMap experiments, we increased their concentration to 30 nM.

### Pseudovirus like Particle Generation

The plasmid pr8ΔEnv.2 was obtained from Addgene, (Plasmid #12263). We generated VLPs as follows: 57 μL of Trans-IT reagent (Mirus) were added to 2 μg of pR8ΔEnv.2, 3 μg of iGFP-GagΔEnv, 1 μg of pcRev and 3 μg of NL4–3 Env to generate HIV-1 VLPs, or 0.5 μg of SARS-CoV-2-Spike [D614G] to generate SARS-CoV-2 VLPs. Importantly, both constructs contain full length Gag and Pol precursor proteins generating fully mature HIV-1 particles. pR8ΔEnv.2 contains WT, unlabeled Gag and HIV iGFP-GagΔEnv contains a GFP tag between the matrix (MA) and capsid subunits (CA). Of note, the GFP sequence is flanked by HIV protease recognition sites allowing dissociation of GFP from CA and MA upon virion maturation. To minimize production of nonfunctional viral particles, expression of labeled Gag has been complemented with unlabeled Gag using a 3:2 ratio. This approach has been demonstrated to retain high infectivity serving as a marker for viral core.^55,56^ To generate mock viruses, no plasmid generating the Env or the Spike protein was added. We then added the mixture to 1.9 mL of OPTIMEM (Gibco) and incubated for 15 min at room temperature. We added the mixture to 18 mL of DMEM with FBS and l-Glut and without antibiotics. We added the medium to HEK-293T cells at 80–90% confluency and collected the supernatant at day 3. We first centrifuged briefly the supernatant (500*g* for 10 min) and filtered the supernatant through a 0.45 μm filter. We concentrated the supernatant with Lenti-X concentrator (Takara) following the manufacturer’s protocol and resuspend it in RPMI. Finally, the VLPs were aliquoted and kept under liquid nitrogen before storing them at −80 °C.

### Sample Preparation for HiDenMap Experiments

We plated ∼50.000 cells on either glass coverslips (#1) coated with PLL (20 ng/mL) for control cells or on 35 mm Glass bottom dish with 10 mm microwell (#1, Cellvis) also coated with PLL. We seeded the cells for 1 h in RPMI without FBS, l-Glut or antibiotics. Viability of the cells was assessed by visual inspection using bright field imaging prior to the experiments. Only well-spread cells with defined dendrites (the normal phenotype of dendritic cells) were carefully chosen for subsequent imaging. Labeling using the single chain-QDs was performed sequentially by diluting 1 μL of DC-SIGN/QD655 and 3 μL of CD44/QD705 in 46 μL of PBS with 6% BSA. We incubated the conjugates and the cells for 5 min. After washing 3 times in RPMI, we took 5 μL of Gal-9/QD605 and 45 μL of PBS diluted in 6% BSA and incubated for 5 min. To avoid removal of the Gal-9 conjugate, we washed only once with RPMI. We then added RPMI to perform the imaging. For the experiments with VLPs (HIV-1, SARS-CoV-2 or mocks) we added the VLPs defrosted and added 10 ng/mL of LPS. The mixture was added to the cells and immediately imaged at 37 °C on the basal membrane for the next 30 min in order to preserve the functionality of the receptors and to avoid internalization.

### Spatiotemporal Autocorrelation Decay Curves

For the analysis on the spatiotemporal autocorrelation functions, we took the HiDenMap generated over 90 s of acquisition and defined regions of interest (ROIs) of 5-by-5 μm. Then, we took all the localizations for the different ROIs of the different proteins and applied an overlapping sliding time window, Δ*t* = 500 ms to temporally separate the localizations. We then computed each autocorrelation curve as follows:
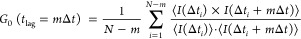
1Here, *I*(Δ*t_i_*) refers to the image of the *i*th temporal window. We then normalized the curve to the first point (*t*_lag_ = 0 s). Finally, we fitted the decay curves from the second point onward until *t*_lag_ = 50 s. We found that the best goodness of the fit consisted on a double exponential decay with a constant term:
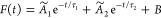
2Finally, once the fitting was performed, we rescaled the amplitudes of the exponential decays as follows to ascertain for the relative weight of the two fitted exponentials:
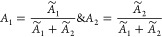
3

See Supporting Information, spatiotemporal autocorrelation decay curves, for more detailed explanations over the temporal window chosen and the autocorrelation fitting procedures.

SPT experiments and diffusion analysis from individual trajectories of DC-SIGN, CD44, and Gal-9. We performed three-color SPT by labeling CD44 and DC-SIGN with conjugated single-chain Abs with QD655 and QD705 respectively, and conjugated biotinylated recombinant Gal-9 with QD565. We performed the imaging at 30 Hz and acquired videos of 90 s. We then performed the tracking using Trackmate (FIJI) and filtered those trajectories shorter than 50 frames (1.5 s). We do not allow reconnection of trajectories in case of a blinking event to avoid false trajectory reconnection that might affect the results. We computed the short-term diffusion coefficient, *D*_1–4_ by fitting the mean-square displacement (MSD) slope for the first 4 time points, in a similar way as described in.^50^

### Determination of Clusters of Localization and Their Radius Using DBSCAN

To quantify the cluster radius of Gal-9, CD44 and DC-SIGN colocalizing with VLPs, we selected 1 μm × 1 μm ROIs of the VLPs as the reference ROI. Then a DBSCAN analysis with ε = 300 nm and the minimum number of points of 10 for Gal-9 and 20 for DC-SIGN and CD44 was computed. To calculate the cluster radius, the MATLAB function polyarea was used.

### Software

For the SPT detection and linking of trajectories we have used ImageJ’s FIJI plugin Trackmate.^46^ All the data have been analyzed using MATLAB R2020a.

## Supporting Information Available

Detailed description of the four-color single molecule sensitive microscope, explanation on the normalization of the localizations of HiDenMaps, detailed explanation on how the spatiotemporal autocorrelation decay curves have been generated and analyzed, description of the multicolor colocalization algorithm (PDF)
CD44 labeled with QD705 and imaged at a frame rate of 30 Hz for 90 s. The imaging is performed on live imDCs under TIR illumination. The scale bar is 10 μm (Video S1) (AVI)
DC-SIGN labeled with QD655 and imaged at a frame rate of 30 Hz for 90 s. The imaging is performed on live imDCs under TIR illumination. The scale bar is 10 μm (Video S2) (AVI)
Gal-9 labeled with QD605 and imaged at a frame rate of 30 Hz for 90 s. The imaging is performed on live imDCs under TIR illumination. The scale bar is 10 μm (Video S3) (AVI)
Overlay of the (Videos S1–S3) keeping the source colors as follows: DC-SIGN(yellow), CD44 (magenta) and Gal-9 (cyan) (Video S4) (AVI)
Three color HiDenMap of DC-SIGN (yellow), CD44 (magenta) and Gal-9(cyan). A time window of 2 s was used to cumulate the localizations of each protein (Video S5) (MP4)
Animation video of four-color single molecule trajectories, with DC-SIGN in yellow, CD44 in magenta, Gal-9 in cyan and, HIV-1 VLP in green. The frame rate for the imaging was 30 Hz and the acquisition was of 90 s. As the video shows, the VLP trajectory appears at later times where a pre-existing nanoplatform of the tripartite proteins was already found (Video S6) (MP4)
Four-color accumulated single-molecule localizations in time using a 2 s windows, showing the successful capture of a HIV-1 VLP by the tripartite proteins, DC-SIGN (yellow), CD44 (magenta) and Gal-9 (cyan). The localizations of each protein have been connected by the clustering algorithm. Scale bar: 1 μm. Note that this video shows a ROI of Video S9 (Video S7) (MP4)
Dynamic HiDenMap showing a VLP vanishing from the field-of-view due to unbinding or because the signal was too low to be detected. The time-window is set to 2 s, total observation time is 90 s. Scale bar is 1 μm. The color-coding is similar as to for Video S4 (Video S8) (MP4)
Dynamic HiDenMap showing a VLP with a transient behavior, i.e., the VLP arrives to the membrane but disengages and disappear. The time-window is set to 2 s, total observation time is 90 s. Scale bar is 1 μm. The color-coding is similar as to for Video S4 (Video S9) (MP4)
Dynamic HiDenMap showing a VLP appearing on the field-of-view, i.e., the VLP arrives at the plasma membrane and remains bound until the end of the acquisition time. The time window is set to 2 s, total observation time is 90 s. Scale bar is 1 μm. The color-coding is similar as to for Video S4 (Video S10) (MP4)
Dynamic HiDenMap showing a VLP with a persistent behavior, i.e., the VLP is visible throughout all the observation time (90 s). The time window is set to 2 s and the scale bar is 1 μm. The color-coding is similar as to for Video S4 (Video S11) (MP4)

## Supplementary Material

nn4c09085_si_001.pdf

nn4c09085_si_002.avi

nn4c09085_si_003.avi

nn4c09085_si_004.avi

nn4c09085_si_005.avi

## Notes

A Modified version of this manuscript has been posted as a preprint in N.M., E.G.-M., J.A.-C., I.C.-A., S.P.-P., M.F.G.-P., J.A.T.-P. Early steps of multireceptor viral interactions dissected by high-density, multicolor single molecule mapping in living cells. 2023. bioRxiv 2023.09.29.560105, 10.1101/2023.09.29.560105.
